# How Possibly Do Leisure and Social Activities Impact Mental Health of Middle-Aged Adults in Japan?: An Evidence from a National Longitudinal Survey

**DOI:** 10.1371/journal.pone.0139777

**Published:** 2015-10-02

**Authors:** Fumi Takeda, Haruko Noguchi, Takafumi Monma, Nanako Tamiya

**Affiliations:** 1 Faculty of Health and Sport Sciences, University of Tsukuba, Ibaraki, Japan; 2 Faculty of Political Science and Economics, Waseda University, Tokyo, Japan; 3 Faculty of Medicine, University of Tsukuba, Ibaraki, Japan; Hamamatsu University School of Medicine, JAPAN

## Abstract

**Objectives:**

This study aimed to investigate longitudinal relations between leisure and social activities and mental health status, considering the presence or absence of other persons in the activity as an additional variable, among middle-aged adults in Japan. This study used nationally representative data in Japan with a five-year follow-up period.

**Methods:**

This study focused on 16,642 middle-aged adults, age 50–59 at baseline, from a population-based, six-year panel survey conducted by the Japanese Ministry of Health, Labour and Welfare. To investigate the relations between two leisure activities (‘hobbies or cultural activities’ and ‘exercise or sports’) and four social activities (‘community events’, ‘support for children’, ‘support for elderly individuals’ and ‘other social activities’) at baseline and mental health status at follow-up, multiple logistic regression analysis was used. We also used multiple logistic regression analysis to investigate the association between ways of participating in these activities (‘by oneself’, ‘with others’, or ‘both’ (both ‘by oneself’ and ‘with others’)) at baseline and mental health status at follow-up.

**Results:**

Involvement in both leisure activity categories, but not in social activities, was significantly and positively related to mental health status in both men and women.

Furthermore, in men, both ‘hobbies or cultural activities’ and ‘exercise or sports’ were significantly related to mental health status only when conducted ‘with others’. In women, the effects of ‘hobbies or cultural activities’ on mental health status were no differences regardless of the ways of participating, while the result of ‘exercise or sports’ was same as that in men.

**Conclusions:**

Leisure activities appear to benefit mental health status among this age group, whereas specific social activities do not. Moreover, participation in leisure activities would be effective especially if others are present. These findings should be useful for preventing the deterioration of mental health status in middle-aged adults in Japan.

## Introduction

Recently, the prevalence rate of mental disorders has been increasing in Japan. The total number of people with mood disorders (including bipolar disorder) was estimated as 958,000 in 2011, of whom 426,000 people were middle-aged adults aged 40 to 64 [1]. Mental health problems are an important contributor to the risk of suicide [2], which was the third most common cause of death (after cancer and heart disease) among middle-aged Japanese adults in 2013 [3].

Growing evidence has indicated that leisure activities (e.g. hobbies, cultural activities, exercise and sports) and social activities (e.g. volunteering and community activities) benefit mental health status among middle-aged and older adults. For example, some cross-sectional and longitudinal studies have reported positive relations between certain types of hobbies or cultural activities, such as going to the cinema or reading newspapers or books, and mental health status among middle-aged and older adults [4, 5]. A cross-sectional study in Japan, Wada et al. reported that regular leisure activity was associated with a reduction in depressive symptoms among workers age 20 to 69 [6], and Wakui et al., using two-year longitudinal data, reported that doing leisure activities at least once per week was inversely related with depression among middle-aged and older caregivers [7].

With regard to exercise and sports activities, considerable evidence exists about their effects on mental health status, and some previous meta-analyses have indicated that exercise interventions were effective in sustaining good mental health status among middle-aged and older adults [8–11].

For social activities, some studies have investigated longitudinal relations between volunteering and mental health status. For example, Li and Ferraro reported that formal voluntary activity was good for mental health status among people aged 60 or older [12]. Potočnik and Sonnentag showed that volunteering improved retirees’ quality of life over a period of two years [13]. In a study of middle-aged Japanese men, those who engaged in more hours of volunteer work had fewer depressive symptoms [14].

Furthermore, the presence of other persons when one is doing these activities can also help to sustain mental health status by providing social relationships. Some meta-analyses have suggested that interventions addressing social relationships can reduce depression [15, 16]. Longitudinal studies with large populations have shown similar findings. One 10-year follow-up study reported that lack of social relationships was a major risk factor for depression among American adults age 25 to 75 [17]. In an 18-year follow-up study, participation in group leisure or social activities was found to benefit the mental health status of older adults [18]. These findings suggest the possibility that doing activities with other persons may have additional positive effects that are not achieved if one engages in leisure activities alone.

However, the effects of leisure and social activities on mental health status among middle-aged adults are still unclear in Japan. No study considering a broad range of leisure and social activities has been conducted, nor has any study investigated whether causal relations between these activities and mental health status are affected by the presence of other persons.

Thus, this study aimed to investigate longitudinal relations between leisure and social activities and mental health status among middle-aged adults, using nationally representative data in Japan, while also considering, as an additional variable, the presence or absence of other persons in those activities.

## Methods

### Study population and procedure

This study used panel data extracted from a nationwide, population-based survey, the ‘Longitudinal Survey of Middle-aged and Elderly Persons (LSMEP)’ conducted once a year on the first Wednesday of November as of 2005 by the Ministry of Health, Labour and Welfare (MHLW) in Japan. Respondents to the survey were extracted randomly through a stratified two-stage sampling. First, 2,515 districts were selected at random from the entire 5,280 districts surveyed by a population-based ‘Comprehensive Survey of the Living Conditions of People on Health and Welfare’ conducted by the MHLW in 2004. Second, 40,877 residents were chosen randomly from those aged 50 to 59 living in each selected district, in proportion to the population size.

In 2005, the first year of the survey, the questionnaires were drop off to the respondents’ homes by enumerators. Then, the enumerators collected the self-completed questionnaire several days later. As of 2006, the method had changed from a “drop-off” to mail survey and so the questionnaire was mailed only to those who had responded to the first survey in 2005. ‘LSMEP’ has not recruited new samples since the first year of survey.

We used data from the first and sixth surveys in 2005–2010. Of the 40,877 people who received a self-administered questionnaire, 34,240 responded to the survey in 2005 (response rate: 83.8%) and these respondents were followed up thereafter. In 2010, the number of respondents decreased to 26,220 (response rate: 64.1%). Out of these, we excluded respondents who had missing values in K6 scale and those who had bad mental health status (K6 total score of 5 points or above). Furthermore, respondents who had some difficulties in activities of daily living were also excluded because they could potentially not do some leisure or social activities, especially exercise or sports. Finally this study used 16,642 respondents (valid response rate was 63.5%).

We obtained an official permission to use ‘LSMEP’ by the MHLW on the basis of Article 32 of the Statistics Act. An ethical review of ‘LSMEP’ was not required, based on the ‘Ethical Guidelines for Epidemiological Research’ of the Japanese government [19].

### Measurements

#### Mental health status

Mental health status was assessed using the Japanese version of the Kessler 6 (K6) scale [20], a screening scale for psychological distress that can effectively discriminate between cases and non-cases of Diagnostic and Statistical Manual of Mental Disorders (DSM-IV) disorders [21]. Respondents answered six items on a 5-point Likert scale, and responses on each item were transformed to scores ranging from 0 to 4 points. A higher total score corresponds to a poorer mental health condition. All respondents were split into two groups, ‘good mental health status’ (scores below 5 points) or ‘bad mental health status’ (5 points or above); the 5-point mark has been identified as the optimal cut-off point for screening mood and anxiety disorders in Japan (100% sensitivity and 68.7% specificity), and it has been used in previous Japanese studies [22, 23]. The Japanese version of the K6 has been validated [20], and the internal consistency reliability (Cronbach’s alpha) of the scale in this study was 0.88.

#### Leisure and social activities

The respondents were asked whether they participated in two types of leisure activities (‘hobbies or cultural activities’ and ‘exercise or sports’) and four types of social activities (‘community events’, ‘support for children’, ‘support for elderly individuals’ and ‘other social activities’) within the past one year from the date of the survey. Those who answered ‘yes’ to each of these question were categorized as ‘active’, and those who answered ‘no’ were categorized ‘inactive’. Those who participated in each of these activities were also asked to indicate in what way they ‘mainly’ participated in the activity (‘by oneself’, ‘with families or friends’, ‘with co-workers (including former co-workers)’, ‘in a neighbourhood community association’ or ‘in a non-profit organization or corporation in the public interest’). For the purposes of this study, respondents were categorized into three groups: ‘by oneself’, ‘with others’ or ‘both’ (both ‘by oneself’ and ‘with others’).

#### Demographic and socioeconomic status

Demographic and socioeconomic status included age (calculated from the month and year of birth), gender, living arrangement (spouse, child or children, father, mother, father-in-law and mother-in-law), job status (employed or unemployed), personal income and family care provision.

#### Chronic diseases

Respondents answered the presence of chronic diseases (diabetes, heart diseases, cerebral stroke, high blood pressure, hyperlipidemia and cancer). They were rated on a dichotomized scale (yes or no).

#### Health behaviour

Health behaviour included smoking status (smoker or non-smoker) and drinking alcohol status (drinker or nondrinker).

### Statistical analysis

We used the multiple imputation by chained equations to handle missing data in this study. Analysis of imputed datasets reduces the potential bias introduced by missing data. This method assumes that data are missing at random, whereby any systematic differences between the missing and observed values can be explained by differences in observed data [24]. Missing values were imputed according to a model consisting of other all variables, and we used multiple imputation to create and analyse 10 multiply imputed datasets. Imputed data were analysed by gender.

At first, in order to investigate the relations between leisure and social activities in the baseline survey and mental health status in the follow-up survey, two kinds of multiple logistic regression models were applied as follows. Model 1 included the six types of leisure and social activities as independent variables, separately (‘hobbies or cultural activities’, ‘exercise or sports’, ‘community events’, ‘support for children’, ‘support for elderly individuals’ and ‘other social activities’); Model 2 included the two types of leisure activities (‘hobbies or cultural activities’ and ‘exercise or sports’) and a summary index which indicates the involvement in at least one of the four social activities (‘community events’, ‘support for children’, ‘support for elderly individuals’ and ‘other social activities’) as independent factors.

Furthermore, we used a multiple logistic regression analysis to investigate the association between the ways of participating in those leisure and social activities (‘inactive’, ‘by oneself’, ‘with others’ or ‘both’) in the baseline survey and mental health status in the follow-up survey.

These multiple logistic regression analyses were adjusted for demographic and socioeconomic status, physical health condition, health behaviour and mental health status at the baseline. The level of significance for all analyses was set at *p* < 0.05. All statistical analyses were performed using IBM SPSS version 23.0.

## Results

Descriptive statistics of the characteristics are shown in Table 1. The K6 score increased significantly from the baseline to the follow-up survey periods in both men and women (using paired t-test: p < 0.001).

**Table 1:** Characteristics of respondents after multiple imputation of missing values.

	Men (n = 8175)				Women (n = 8467)				
	Mean (SE)		n (%)		Mean (SE)		n (%)		*p*
**Demographic and socioeconomic status**									
Age (years)	54.76	(0.03)			54.73	(0.03)			0.446^a^
Living arrangement									
Spouse (Presence)			7193	(88.0)			7189	(84.9)	<0.001^b^
Child(ren) (Presence)			5206	(63.7)			5213	(61.6)	0.005^b^
Father (Presence)			874	(10.7)			271	(3.2)	<0.001^b^
Mother (Presence)			1948	(23.8)			680	(8.0)	<0.001^b^
Father-in-law (Presence)			208	(2.5)			526	(6.2)	<0.001^b^
Mother-in-law (Presence)			464	(5.7)			1317	(15.5)	<0.001^b^
Job status (Employment)			7858	(96.1)			6051	(71.5)	<0.001^b^
Personal income (thousand yen)	52.13	(0.80)			30.65	(0.60)			<0.001^a^
Family care provision (Yes)			435	(5.3)			759	(9.0)	<0.001^a^
**Chronic diseases**									
Diabetes (Presence)			662	(8.1)			331	(3.9)	<0.001^b^
Heart diseases (Presence)			238	(2.9)			100	(1.2)	<0.001^b^
Cerebral stroke (Presence)			72	(0.9)			50	(0.6)	0.028^b^
High blood pressure (Presence)			1504	(18.4)			1247	(14.7)	<0.001^b^
Hyperlipidemia (Presence)			739	(9.0)			725	(8.6)	0.277^b^
Cancer (Presence)			81	(1.0)			126	(1.5)	0.004^b^
**Health behaviour**									
Smoking status (Smoker)			3774	(46.2)			886	(10.5)	<0.001^b^
Drinking alcohol status (Drinker)			2033	(24.9)			5874	(69.4)	<0.001^b^
**Leisure and social activities**									
Hobbies or cultural activities (Active)			4784	(58.5)			5533	(65.3)	<0.001^b^
Exercise or sports (Active)			3971	(48.6)			3835	(45.3)	<0.001^b^
Community events (Active)			2490	(30.5)			2494	(29.5)	0.158^b^
Support for children (Active)			436	(5.3)			558	(6.6)	0.001^b^
Support for elderly individuals (Active)			476	(5.8)			761	(9.0)	<0.001^b^
Other social activities (Active)			909	(11.1)			1038	(12.3)	0.022^b^
**Mental health status**									
Baseline	0.98	(0.02)			1.14	(0.02)			<0.001^a^
Follow-up	2.06	(0.03)			2.43	(0.04)			<0.001^a^
Bad mental health at follow-up			1353	(16.6)			1677	(19.8)	<0.001^b^

^a^ Independent t-test

^b^ Chi-square test.

One thousand three hundred fifty three (16.6%) of men, and 1,677 (19.8%) of women were categorized into the group of bad mental health status in the follow-up surveys. The proportion of people who had bad mental health status in women was larger than that in men.

The number of respondents who participated in each of leisure and social activities at the baseline was as follows: 4,784 (58.5%) of men and 5,533 (65.3%) of women in ‘hobbies or cultural activities’, 3,971 (48.6%) of men and 3,855 (45.3%) of women in ‘exercise or sports’, 2,490 (30.5%) of men and 2,494 (29.5%) of women in ‘community events’, 436 (5.3%) of men and 558 (6.6%) of women in ‘support for children’, 476 (5.8%) of men and 761 (9.0%) of women in ‘support for elderly individuals’ and 909 (11.1%) of men and 1038 (12.3%) of women in ‘other social activities’. The proportion of people who participated in ‘hobbies or cultural activities’, ‘support for children’, ‘support for elderly individuals’ and ‘other social activities’ in women was larger than that in men, whereas that of people who participated in ‘exercise or sports’ in men larger than that in women.

Table 2 shows the results of multiple logistic regression analyses. In regard to men, the result of Model 1 showed that ‘hobbies or cultural activities’ (OR 0.85, 95% CI 0.74–0.98, *p* < 0.05) and ‘exercise or sports’ (OR 0.85, 95% CI 0.74–0.98, *p* < 0.05) were significantly related to mental health status at the follow-up period. Model 2 also showed a similar result to Model 1, such that ‘hobbies or cultural activities’ (OR 0.84, 95% CI 0.73–0.98, *p* < 0.05) and ‘exercise or sports’ (OR 0.86, 95% CI 0.74–0.99, *p* < 0.05) had a significant relation with mental health status in the follow-up survey.

**Table 2:** Multiple logistic regression analyses for the relations between leisure and social activities and mental health status at the follow-up period.

		Model 1^a^			Model 2^a^		
		AOR	95% CI	*p*	AOR	95% CI	*p*
**Men**							
Hobbies or cultural activities	Active (ref. Inactive)	0.85	0.74–0.98	0.023	0.84	0.73–0.98	0.028
Exercise or sports	Active (ref. Inactive)	0.85	0.74–0.98	0.029	0.86	0.74–0.99	0.034
Community events	Active (ref. Inactive)	1.01	0.87–1.18	0.855			
Support for children	Active (ref. Inactive)	0.89	0.60–1.31	0.550			
Support for elderly individuals	Active (ref. Inactive)	1.32	0.94–1.87	0.110			
Other social activities	Active (ref. Inactive)	0.85	0.65–1.13	0.263			
Social activities	Active (ref. Inactive)				0.98	0.86–1.12	0.740
**Women**							
Hobbies or cultural activities	Active (ref. Inactive)	0.72	0.63–0.83	0.000	0.71	0.61–0.84	0.000
Exercise or sports	Active (ref. Inactive)	0.88	0.77–1.00	0.042	0.88	0.78–1.01	0.069
Community events	Active (ref. Inactive)	0.96	0.81–1.13	0.578			
Support for children	Active (ref. Inactive)	1.18	0.86–1.62	0.294			
Support for elderly individuals	Active (ref. Inactive)	1.16	0.86–1.58	0.320			
Other social activities	Active (ref. Inactive)	0.88	0.63–1.22	0.420			
Social activities	Active (ref. Inactive)				1.01	0.90–1.14	0.852

^a^ Adjusted for demographic and socioeconomic status, physical health condition, chronic diseases and mental health status at the baseline.

AOR: Adjusted odds ratio; CI: Confidence interval.

In regard to women, the result of Model 1 showed that ‘hobbies or cultural activities’ (OR 0.72, 95% CI 0.63–0.83, p < 0.001) and ‘exercise or sports’ (OR 0.88, 95% CI 0.77–1.00, p < 0.05) significantly associated with mental health status, as was the case with men. By contrast, Model 2 showed that ‘hobbies or cultural activities’ were significantly related only to mental health status at the follow-up period (OR 0.71, 95% CI 0.61–0.84, p < 0.001).

Furthermore, the relations between the ways of participating in ‘hobbies or cultural activities’ or ‘exercise or sports’ and mental health status were investigated in both men and women. Table 3 shows the results when the ‘in active’ category as reference was selected. Regarding men, both ‘hobbies or cultural activities’ and ‘exercise or sports’ were significantly related to mental health status only when conducted ‘with others’ (‘hobbies or cultural activities’: OR 0.83, 95% CI 0.71–0.97, *p* < 0.05; ‘exercise or sports’: OR 0.84, 95% CI 0.73–0.98, *p* < 0.05). Regarding women, ‘exercise or sports’ was significantly related to mental health status only when conducted ‘with others’ (OR 0.86, 95% CI 0.75–0.99, *p* < 0.05), whereas significant ORs for ‘hobbies or cultural activities’ were observed in all of three categories (‘by oneself’: OR 0.74, 95% CI 0.62–0.90, *p* < 0.01; ‘with others”: OR 0.76, 95% CI 0.67–0.87, *p* < 0.001; ‘Both’: OR 0.55, 95% CI 0.33–0.93, *p* < 0.05).

**Table 3:** Multiple logistic regression analyses for the relations between the way of participation and mental health status at the follow-up periods.

		Men				Women			
		n	AOR^a^	95% CI	*p*	n	AOR^a^	95% CI	*p*
Hobbies or cultural activities (ref: Inactive)	By oneself	1350	0.96	0.81–1.15	0.683	1107	0.74	0.62–0.90	0.002
	With others	3317	0.83	0.71–0.97	0.018	4291	0.76	0.67–0.87	0.000
	Both	116	0.85	0.39–1.86	0.689	135	0.55	0.33–0.93	0.024
Exercise or sports (ref: Inactive)	By oneself	1283	0.93	0.77–1.11	0.408	1310	0.89	0.75–1.05	0.158
	With others	2560	0.84	0.73–0.98	0.030	2405	0.86	0.75–0.99	0.039
	Both	126	0.79	0.33–1.89	0.596	120	0.97	0.59–1.56	0.887

^a^ Adjusted for demographic and socioeconomic status, physical health condition, health behaviour and mental health status at baseline.

AOR: Adjusted odds ratio, CI: confidence interval.

Additionally, when the ‘with others’ category as reference was selected, the significant OR was observed only in the ‘inactive’ category (‘hobbies or cultural activities’: OR 1.21, 95%CI 1.03–1.41, *p* < 0.05 in men, OR 1.32, 95%CI 1.16–1.50, *p* < 0.01 in women; ‘exercise or sports’ OR 1.18, 95%CI 1.02–1.38, *p* < 0.05 in men, OR 1.16, 95%CI 1.01–1.34, *p* < 0.05 in women).

## Discussion

The main objective of this study was to investigate relations between leisure and social activities and mental health status among middle-aged adults in Japan using nationally representative data. The results show that participation in leisure activities such as ‘hobbies or cultural activities’ and ‘exercise or sports’ at the baseline would be positively related to mental health status at the period after five-year follow-up in both men and women. Therefore, that is consistent to some previous cross-sectional and longitudinal studies which have reported relations between involvement in hobbies or cultural activities and mental health status in middle-aged and older adults [4–7]. Moreover, some meta-analyses have found physical activity interventions to be effective in sustaining mental health status within the same age cohort [8–11]. Leisure activities could play a role in benefitting overall well-being and providing a buffer against stress. This benefit may occur by promoting a variety of social and physical resources that enable individuals to feel refreshed and to cope adequately with stress [25, 26].

In contrast, this study observed no benefits of the participation in social activities at the baseline on mental health status in the follow-up survey in both men and women. Several previous studies reported the longitudinal relations between social activities and mental health status. Li and Ferraro found that volunteer work had beneficial effects on mental health status among older adults [12]. A previous study in Japan suggested that volunteer work was associated with reduced depressive symptoms among adults in later middle age, even after controlling for pre-existing depressive symptoms, socioeconomic factors and physical health [14]. However, these studies did not control the influence of activities other than social activities. Our findings showed no longitudinal relation between social activities and mental health status when the effects of leisure activities were considered.

One previous study reported that social activities were associated with longitudinal changes in mental health status even after considering the influence of leisure activities [27]; however, respondents in that study were much older than that those in our study. Because the perceived value of life tends to decrease gradually with age [28], maintaining social activities may be especially important to sustain good mental health status among older adults. However, because our respondents were still relatively young in their fifties, and 96.4% of men and 71.5% of women had a job at the baseline period, almost all of them had other forms of regular social involvement. This could be one reason why our results indicate that leisure activities contribute to mental health status in middle-aged adults, whereas specific social activities do not.

Furthermore, this study investigated the relations between the presence of other persons in leisure activities and mental health status. Our results showed that hobbies or cultural activities in men and exercise or sports in both men and women would reduce such risk only when conducted with others, whereas hobbies or cultural activities in women might have effects on mental health regardless of the presence of others.

These results suggested that social relationships through leisure activities would be the key factor of preventing the deterioration of mental health status regardless of gender differences. Some previous studies have suggested that the improvements in mental health status following exercise or sports could be partially because of the social relations that can be experienced through participating in these activities with others [29, 30]. However, in this study, no relation existed between doing exercise or sports by oneself and mental health status. The previous study in Japan suggested that, even when exercise was performed once a week or more, incident of functional disability might be better prevented if the person participated in a sports organization than if they did not [31]. Our result suggests that the psychological effects of social relations may be especially needed in not only the case of exercise or sports, but also the case of hobbies or cultural activities. On the other hand, we found no significant difference between ‘by oneself’ or ‘both’ category and ‘with others’ category. Thus, the effect of the presence of others may need to be carefully considered.

This is the first study to show a longitudinal relation between leisure and social activities and mental health status among middle-aged adults in Japan. The study has several particular strengths. First, it used a good set of nationally representative data. Second, unlike previous studies of middle-aged adults in Japan, our study encompassed a wide range of leisure and social activities. Finally, our study indicated further details about the appropriate way of doing leisure activities to maintain good mental health status while approaching old age (i.e. whether it is effective to do these activities alone or only with others).

The study also has several limitations. First of all, this study might not completely identify the pure effects of the participation into leisure and social activities on mental health status, because the data in the baseline period must suffer from reversed causality problem between the participation and mental health status. In another word, those who are in better mental health status are more likely to involve into various social activities. Second, although the multiple imputation was used to try to reduce impact of missing variables, our study would still have some selection bias. About 6,000 people did not respond a questionnaire in the baseline survey, and almost 8,000 respondents were dropped out in the follow-up study. Thus, generalization of the results in our study should be done carefully. Third, the participation in leisure and social activities was indicated on a dichotomized scale, and thus, the frequency or variety of participation was not assessed. Forth, questionnaires about leisure and social activities were self-reported and retrospective, rendering them somewhat inaccurate. Finally, ‘LSMEP’ excluded patients in hospitals and clinics and residents of long-term elderly care facilities. These people might have a higher-than-average rate of bad mental health status and might not have been likely to engage in leisure and social activities. Therefore, possibly, the positive relations between leisure and social activities and mental health status were underestimated.

In conclusion, this study indicates that leisure activities might contribute to good mental health status among middle-aged adults in Japan, whereas social activities would provide no mental health status benefit. Moreover, participation in leisure activities would be effective especially if others are present. These findings may be useful for preventing the deterioration of mental health status in middle-aged adults in Japan.
